# Harnessing Cationic Bilosomes to Create a Green Light-Triggered Nanoplatform for Skin Melanoma Treatment

**DOI:** 10.2147/NSA.S531026

**Published:** 2025-09-22

**Authors:** Ewelina Waglewska, Julita Kulbacka, Urszula Bazylińska

**Affiliations:** 1 Department of Physical and Quantum Chemistry, Faculty of Chemistry, Wroclaw University of Science and Technology, Wroclaw, Poland; 2 Department of Molecular and Cellular Biology, Faculty of Pharmacy, Wroclaw Medical University, Wroclaw, Poland; 3 State Research Institute Centre for Innovative Medicine, Department of Immunology, Vilnius, Lithuania

**Keywords:** lipid-based nanovesicular systems, bile salts, Rose Bengal, astaxanthin, antitumor activity, phyto-photodynamic therapy

## Abstract

**Background:**

Vesicular drug delivery systems, including bilosome-based nanoparticles containing bile salts, have revolutionized the field of colloid chemistry, nanomedicine, and nanobiotechnology. Due to their versatility and adaptability to various applications, they have gained considerable attention among researchers, thus offering a promising pathway to achieve effective and targeted delivery of miscellaneous drugs.

**Purpose:**

This study presents a novel class of positively charged bilosomes with surface-associated poly(ethylene glycol) (PEG)-lipid, co-entrapped the anionic xanthene dye (Rose Bengal), and natural carotenoid pigment derived from the mold *Blakeslea trispora* (astaxanthin), as a safe and effective transdermal drug delivery system.

**Methods:**

Bilosomal nanosystems were prepared using thin film hydration combined with sonication. The physicochemical properties of the vesicles were characterized, including particle size, zeta potential, entrapment efficiency, and morphology. Cellular uptake, cyto- and phototoxicity experiments were investigated in vitro against human melanoma cancer cells.

**Results:**

The multidrug bilosome formulation exhibited a particle size of less than 100 nm and a zeta potential of more than +40 mV, indicating beneficial properties for potential transdermal administration. In vitro biological experiments have shown remarkable antitumor efficacy against human skin epithelial (A375) and malignant (Me45) melanoma cell lines. After irradiating the samples with green light at a wavelength of 520–560 nm (10 J/cm^2^ of total light dose), we observed a significant decrease in mitochondrial metabolic activity, ie, a reduction in cell viability below 30% compared to the control. Higher phototherapeutic activity, in contrast to the administration of non-encapsulated active agents, indicates shared synergistic effects through the simultaneous action of advanced bilosome-derived nanophotosensitizers and phyto-photodynamic therapy.

**Conclusion:**

Our encouraging results provide new potential candidates for preclinical development in innovative photodynamic therapy targeting melanoma and also pave the way for future therapeutic strategies with broad applications in many biological fields.

## Graphical Abstract

**Figure:**
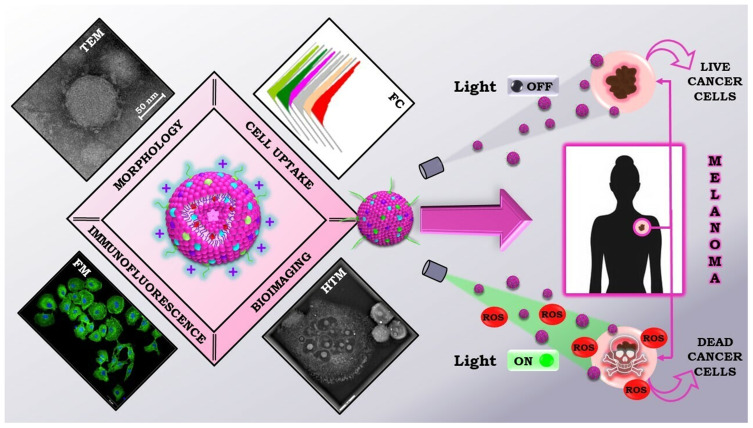

## Introduction

In recent years, nanotechnology has played a crucial role in designing intelligent colloidal materials responsible for precise drug delivery to pathological tissues, thus revealing a new dimension of treatment in the selected medical fields. These drug delivery systems (DDSs) can encapsulate, protect, and release pharmaceuticals at the target site, thereby improving their bioavailability and maximizing therapeutic effects. DDSs can also reduce the side effects associated with some conventional drug formulations. Finally, they provide controlled and targeted delivery of active pharmaceutical ingredients (APIs) via various administration routes.^1–3^

Transdermal drug delivery has several advantages, including allowing APIs to be directly accessed through the skin surface into the bloodstream, bypassing the hepatic first-pass effect, and the possibility of self-administration, non-invasiveness, and high patient compliance. However, the effective interaction of DDS with the skin and the subsequent transdermal diffusion of pharmaceuticals depends on several crucial factors, ie, the characteristics of the systems under development and the nature of the charges and structural conditions inside the skin. Accordingly, lipid vesicles, as one of the most versatile types of DDS, can be divided into conventional and newly deformable liposomes.^4^ Conventional vesicles consist of natural or synthetic amphiphilic molecules with a hydrophilic head and hydrophobic tail (phospholipids, PLs) with added cholesterol (Chol). In this case, their rigid structure and insufficient stability prevent them from penetrating the deep layers of the skin.^5^ Advanced vesicles (bilosomes) composed of lipid bilayer softeners (ie, bile salts, BS) have become an excellent alternative to the commonly used conventional liposomes. The co-existence of these steroidal biosurfactants in the bilosome structure leads not only to increased flexibility and deformability but also to improved colloidal stability and permeability of the system through biological membranes due to its high biocompatibility and ability to interact with phospholipids. It is also worth noting that some bile salts can increase membrane permeability and improve the penetration of the nanocarrier (NC) into the desired skin layers, followed by interactions with keratin fibers, leading to damage to dead cells (corneocytes) or modification of proteins present in the lipid bilayer of the *stratum corneum* (SC).^6^,^7^ This phenomenon is necessary but not limited to the only requirement for transdermal application.^8–10^ Another significant factor for this drug delivery route is the surface charge of the nanocarriers. SC lipid layers contain a high amount of negatively charged lipids, indicating that the skin can act as a negatively charged cell membrane. Unlike neutral and negatively modified carriers, positively charged nanosystems can promote more effective permeation of APIs due to their strong electrostatic attraction to the skin surface.^4^,^11^,^12^ Therefore, modifying the surface structure by introducing cationic components (including biocompatible lipids) into the formulation can increase their therapeutic efficacy. Conducting intensive research in this area may also enable significant future advances in the applications of positively charged NCs in transdermal therapies.^12^

Between many different compounds that can be incorporated into NCs, photosensitizing agents are attracting increasing attention among researchers due to the encouraging results of photodynamic therapy (PDT) in treating various types of cancer, including FDA-approved for the treatment of non-melanoma skin cancers, esophageal (throat) cancer, or non-small cell lung cancer.^13–16^ Compared to standard therapeutic approaches, PDT works by selectively destroying only target cells without damaging healthy tissues, making this method primarily characterized by a non-invasive nature, minimal cytotoxicity, remote controllability, low incidence of side effects, and short recovery period for the patient. This treatment relies on photosensitizer (PS) application, which, when exposed to light of the appropriate wavelength, transfers light energy directly to surrounding molecules to produce reactive oxygen species (ROS), such as highly potent singlet oxygen (^1^O_2_). So, choosing the optimal light wavelengths, requisite oxygen supply, and allowable PSs are decisive factors for achieving the desired PDT result.^17^,^18^ Rose Bengal (RB), chemically the 3′, 4′, 5′, 6′ -tetrachloro-2,4,5,7-tetraiodo-fluorescein is a fluorescent dye belonging to the xanthene family, considered among the most efficient second-generation PSs with highly water solubility. The presence of high atomic number halogen atoms (ie, chlorine and, in particular, iodine) in the structure of xanthene rings, providing the so-called heavy atom effect, allows promoting intersystem crossing in the excited-state RB after irradiation with visible green light (λ_max_ = 546 nm in water) due to enhanced spin-orbit coupling. This phenomenon leads to an increased population of triplet states and, thus, the production of ROS. In this connection, the high singlet oxygen quantum yield of the RB(Φ_Δ_^1^O_2_ ~ 0.75) is crucial to its usefulness in photochemical and photodynamic processes.^19–21^ Despite its demonstrated properties and excellent therapeutic potential, the clinical application of RB is limited due to its unfavorable biopharmaceutical profile. Its anionic nature, as well as high molecular weight (equal to 1017.64 g/mol for the RB disodium salt), prevents it from freely permeating cell membranes, which negatively affects cellular uptake, proper cellular accumulation, and biodistribution.^22^,^23^

Recently, increasing attention has been paid to the potential use of NCs in combined anticancer therapy (ie, chemo-radio, chemo-phyto, or chemo-immuno), providing synergistic efficacy and minimizing side effects, primarily due to its multidirectional impact on tumors, while preventing multidrug resistance through various biological mechanisms. So, due to their numerous pharmacological properties, bioactive compounds of plant origin are increasingly being used by researchers as anticancer boosters.^24^,^25^ Prominent among them is a non-flavonoid polyphenol (astaxanthin, AST), which performs an essential function in treating melanoma - the most invasive skin cancer with the highest risk of death. It can inhibit the growth of human melanoma by reducing the number of metastases and inducing apoptosis by, among other things, down-regulating the expression of anti-apoptotic proteins (Bcl-2, p-Bad, and survivin) and increasing the expression of the pro-apoptotic ones (Bax/Bad and PARP). On the other hand, AST treatment can reduce melanoma cell migration by inhibiting the expression of matrix metalloproteinase (MMP) 1, 2, and 9, as well as promote wound re-epithelialization, thereby increasing the number and recruitment of keratinocytes in a dose-dependent manner.^26^,^27^ Accordingly, phytotherapeutics can be investigated for their potential synergistic role with other chemical, light-activated molecules, leading to the the development of modern carrier-assisted phyto-PDT for the treatment of various conditions, with a remarkable focus on melanoma skin cancer.^28^

**Figure 1:**
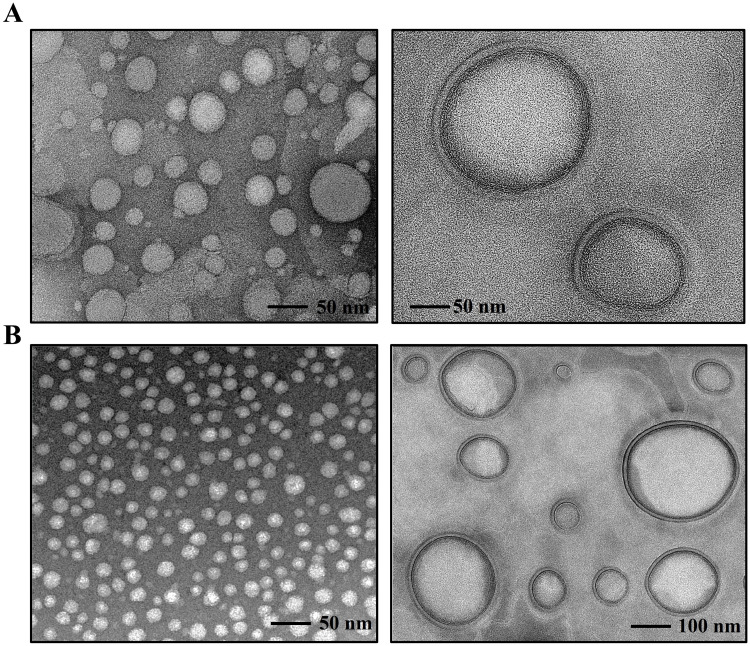
TEM micrographs of empty bilosomes (**A**) and RB/AST-loaded bilosomes (**B**).

**Scheme 1:**
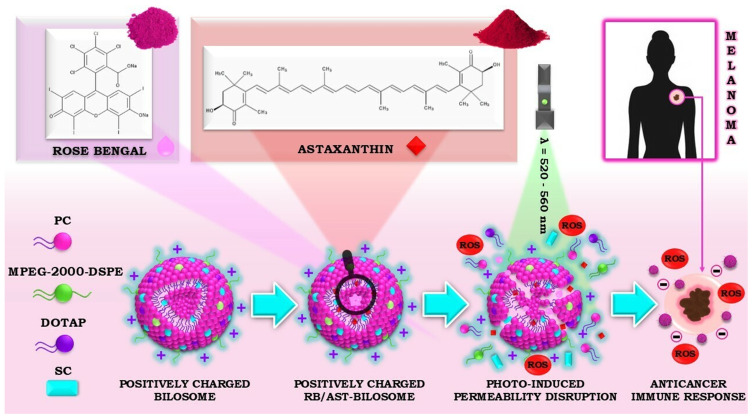
Schematic representation of the general idea of the research involving the colloidal system encapsulating RB and AST as an innovative nanophotosensitizer of bilosomal origin for modified photodynamic therapy targeting human melanoma.

Prompted by these findings, we designed and made an original positively charged bilosomes based on phosphatidylcholine (PC), 1,2-dioleoyl-3-trimethylammonium-propane, chloride (DOTAP) modified with sodium cholate (SC) and N-(carbonyl-methoxypolyethylene glycol-2000)-1,2-distearoyl-sn-glycero-3-phosphoethanolamine, sodium salt (MPEG-2000-DSPE) (see Figure 1)

designed to co-encapsulate two active compounds (Scheme 1). Then, we investigated several basic physicochemical properties of the resulting nanoplatform, including size, surface charge, morphology, and encapsulation efficiency of the co-loaded cargo. The crucial step was to evaluate the nanocarrier’s ability to generate singlet oxygen by spectrophotometrically monitoring the photooxidation of the chemical probe 9,10-anthracenediyl-bis(methylene)dimalonic acid (ABMDMA) and test the developed nanoplatform in vitro against human melanoma cell lines (A375 and Me45) to confirm its efficacy in phyto-PDT. There are reports of the use of bilosomes (the vast majority of negatively charged) in the treatment of many diseases.^29–32^ However, we are not aware of any published work on positively charged “stealth” bilosomes used to co-encapsulate two biologically active ingredients of different solubilities targeting melanoma. This research represents the first and fundamental report on the unique therapeutic efficacy of a bilosomal nanosystem combining the high photodynamic activity of a second-generation photosensitizer (Rose Bengal, RB) and supported by a natural anticancer agent (astaxanthin, AST).

## Materials and Methods

### Materials

Biosurfactants: L-α-Phosphatidylcholine (PC) from egg yolk (~60%) and anionic sodium cholate (SC) hydrate (≥97%; dried material, NT) were purchased from Sigma-Aldrich (Poznań, Poland). PEGylated phospholipid product: *N*-(carbonyl-methoxypolyethylene glycol-2000)-1,2-distearoyl-*sn*-glycero-3-phosphoethanolamine, sodium salt (MPEG-2000-DSPE) and cationic lipid: 1,2-dioleoyl-3-trimethylammonium-propane chloride (DOTAP) were generous gifts from Lipoid GmbH (Ludwigshafen, Germany) andstored at −20°C until used. All the chemical structures of the compounds used to prepare the bilosomal system are shown in Figure 1. Xanthene-based hydrophilic photosensitizer - Rose Bengal (RB, dye content 95%) and xanthophyll carotenoid – astaxanthin from *Blakeslea trispora* (AST, ≥97%) were applied as a model hybrid cargo (Sigma-Aldrich, Poznań, Poland). 9,10-anthracenediyl-bis(methylene)dimalonic acid (ABMDMA) and thiazolyl blue tetrazolium bromide (MTT; ≥97.5%) were purchased from Sigma Aldrich (Poznań, Poland). All organic solvents - chloroform (CHCl_3_), tetrahydrofuran (THF), and dimethyl sulfoxide (DMSO) were bought from Avantor Performance Materials Poland S.A. (Gliwice, Poland). The aqueous solutions were prepared using distilled water purified through a system HLP Smart Hydrolab (Labsystem S.C., Kraków, Poland). All chemical compounds were applied with no further purification.

HaCaT (immortalized human epidermal keratinocytes, ATCC PCS‑200‑013, passages 15–25) and A375 cells (ATCC CRL‑1619, passages 7–15) were purchased from ATCC^®^ (LGC Standards, Poland). The Me45 cell line (passages 10–20) is a human primary malignant melanoma cell line that was originally established in 1997 from a lymph node metastasis of a 35-year-old female patient at the Radiobiology Department of the Oncology Centre in Gliwice, Poland. The donor provided written informed consent, and the appropriate Hospital Committee approved the procedure before its commencement. The resulting cells, used for all in vitro biological studies, were maintained in standard vent‑cap flasks (Falcon^®^ Cell Culture Flasks) with an area equal to 25 or 75 cm^2^ in Dulbecco’s Modified Eagle’s Medium (high glucose; DMEM, IITD, Wroclaw, Poland) to promote cell growth. The medium to be completed was supplemented with 10% fetal bovine serum (FBS, Sigma-Aldrich, Poznań, Poland) and antibiotics: 50 μg/mL penicillin and streptomycin (Sigma-Aldrich, Poznań, Poland). The cultures were incubated under standard environmental conditions crucial for cell growth: a temperature of 37°C and a humidified atmosphere with 5% CO_2_. When necessary, the cells were detached by trypsinization using Trypsin-EDTA solution, neutralized with complete cell culture medium (DMEM containing FBS), and further analyzed in subsequent experiments. All experiments were conducted in accordance with the guidelines of the Bioethics Committee at Wroclaw Medical University, and the Hospital Committee of the Oncology Centre in Gliwice (Me45 cell line), adheringto the principles of the Declaration of Helsinki.

### Synthesis of Polyethylene Glycol (PEG)-Enriched Positively Charged Bilosomes

The lipid film hydration technique (otherwise Bangham method) followed by sonication was employed to create nanomedicine-co-loaded positively charged bilosomes. In brief, a mixture of lipids and astaxanthin was completely dissolved in 3 mL of CHCl_3_ at a total concentration of 6 mg/mL PC, 4 mg/mL DOTAP, 0.5 mg/mL MPEG-2000-DSPE, and 0.05 mg/mL AST, and were lightly stirred by shaking a 10 mL round bottom flask. The organic solvent was evaporated by Heidolph Hei-VAP Value Digital Rotary Evaporator (Schwabach, Germany) at 45°C while controlling the pressure until a lipid film formed on the walls of the flask. Simultaneously, an aqueous-phase solution containing RB and SC at concentrations of 0.15 mg/mL and 1 mg/mL, respectively, was prepared and used to hydrate the resulting thin lipid film for about 3 hours. After hydration, thebilosomal dispersion was sonicated in a bath sonicator (Bandelin, Sonorex Digitec DT 100H, Germany) for approximately 3 minutes. Non-encapsulated bilosomes (without RB and AST) were similarly prepared for reference, which was also accurately described in our earlier report.^33^ Such prepared nanocarriers were stored at 4°C and used for further experiments within 30 days.

### Characterization Methods of Colloidal Dispersions

#### Dynamic Light Scattering (DLS) Analysis

The hydrodynamic diameter and size distribution of both empty and co-loaded positively charged bilosomes were measured in distilled water using a dynamic light scattering (DLS) method with a Zetasizer Pro Blue series from Malvern Panalytical company (Worcestershire, UK), equipped with a 4.0 mW He-Ne laser operating at 633 nm with an avalanche photodiode detector at a 173 ° detection angle. The “general purpose” model, appropriate for most dispersions, was the data processing analysis model used in the ZS XPLORER software. In addition, the “liposomes” were applied as a measurement material (from the set of materials available in the software) with a refractive index of 1.45 and an absorption of 0.001. The colloidal bilosomes dispersion was transferred first to a disposable polystyrene cuvette and then placed in the apparatus. All measurements were conducted at 25°C, and automatic optimization of beam focusing and attenuation was used for each measured sample. Values were recorded as the mean diameter (Z-average) and polydispersity index (PdI) of the nanovesicles’ samples, which were ultimately reported as the mean of three runs with at least ten measurements for three independent experiments (n = 3, means ± SD).

#### Electrophoretic Light Scattering (ELS) Analysis

Zeta potential analysis, indicating sample stability and tendency to aggregate, was conducted in 0.25% (w/v) saline solution (pH 7.4, conductivity 2.0 mS/cm) with the Malvern Zetasizer Pro (Blue) apparatus (Malvern Instruments, Worcestershire, UK). The measurements were performed in a disposable folded capillary zeta cell (DTS1070) at a room temperature of 25°C. Each value of bilosomes’ ζ-potential (mV), estimated from the electrophoretic mobility applying to the Smoluchowski approximation, were designated as the mean of three successive instrument runs with at least ten to twenty measurements. Each data set represents three independent experiments for each formulation (n = 3, means ± SD).

#### TEM Microscopy

The morphology of the positively charged bilosomal and RB/AST-co-loaded bilosomes models was evaluated by a transmission electron microscope (TEM). After 10-fold dilutions of particular formulations, the samples were placed on the carbon-coated support copper TEM grids (~ 10 µL), allowed to dry completely at room temperature for 1 hour, and were finally analyzed using an FEI Tecnai G2 20 X-TWIN transmission electron microscope (Hillsboro, OR, USA).

#### Ultraviolet-Visible (UV-Vis) Spectrophotometric Measurements

The encapsulation efficiency (EE) and drug loading (DL) capacity of Rose Bengal (RB) and astaxanthin (AST) in bilosomal formulation were determined using a high-performance UV-Vis double-beam spectrophotometer (HALO DB-20S, Dynamica Scientific Ltd., Livingston, UK). Firstly, the standard curves for the above active compounds were obtained by detecting standard samples of RB and AST at different concentrations in a mixture of THF : H_2_O (3:1) at the corresponding wavelengths of maximum absorbance (λ_RB_ = 561 nm and λ_AST_ = 483 nm). Afterward, the dialysis process was employed to remove the entirely non-encapsulated substances using dialysis bags purchased from Carl Roth (cut-off: 14,000 Da, Germany). At last, the positively charged nanostructures were disrupted with THF: H_2_O, followed by the UV-Vis measurements performed in the quartz cuvette with a 1 cm path length. EE and DL were calculated as follows:

(1) $${\rm EE} = {{amount \;of \;the \;pharmaceutical \;agent \;entrapped \;in \;bilosomes } \over {initial \;amount \;of \;the\ pharmaceutical \;agent \;added \;in \;bilosomes}} \times 100\rm {\%}$$

(2) $${\rm DL} = {{amount \;of \;the \;pharmaceutical \;agent \;entrapped \;in \;bilosomes } \over {total \;amount \;of \;bilosomes}} \times 100\rm {\%}$$

### Mechanism of Singlet Oxygen Generation by the ABMDMA Assays

The efficiency of producing a pivotal cytotoxic factor (ie, singlet oxygen, ^1^O_2_) against cancer cells in PDT was detected chemically using the 9,10-anthracenediyl-bis(methylene)dimalonic acid (ABMDMA) probe, which converts to its non-fluorescent endoperoxide in the presence of ^1^O_2_. Based on the previously used procedure,^34^ for this purpose, we used a 0.15 mM stock solution of the sodium salt of ABMDMA prepared in DMSO, which was then mixed with native RB or RB/AST-loaded positively charged bilosomes, obtaining the final photosensitizer concentration in the range of 0.5 µM to 12.5 µM. The conducted reactions were monitored spectrophotometrically for 40 minutes at specified intervals, recording the decrease in optical density at 379 nm (λ_max_ ABMDMA). All irradiation studies were performed using an OPTEL Fibre Illuminator (Opole, Poland) equipped with a polarising plate and a green filter (λ_max_ 520–560 nm).

### Exploring Biological Activities and In Vitro Anticancer Effects of Novel Hybrid Nanoplatform

#### Comparative Assessment of Cellular Active Compounds Uptake

The ability of melanoma cells (A375 and Me45) to internalize and uptake free and encapsulated RB and AST in the developed bilosomes was evaluated by flow cytometry (CyFlow Cube 6, Sysmex, Poland). For flow cytometry uptake studies, the cells were trypsinised to generate single-cell suspensions; parallel monolayer experiments were performed on cultures that were 90% confluent to mimic an intact epidermal sheet. The dispersion of positively charged bilosomes was added with the final RB concentration equal to 1 µM and 2 µM. Then, the cells were incubated in a humidified atmosphere containing 5% CO_2_ at 37°C for 24 hours. After that time, the cells were washed in PBS, trypsinized, and resuspended in 0.5 mL of PBS. The fluorescence of RB with AST was assessed with an FL-3-H detector (RG 630 nm). Every sample was analyzed in at least three replicates (10^4^ cells/sample were measured each time). The data was collected and analyzed using CyView software (Sysmex, Poland).

#### Immunofluorescence Cell Staining and Holotomographic (HTM) Morphology Imaging

An Olympus BX53 fluorescent microscope (FM, Evident Europe GmbH, Warsaw, Poland) was used to visualize the actin filaments and nuclei of the cells studied. The A375 and Me45 cells were seeded directly on 18 mm diameter round microscope coverslips (Thermo Fisher Scientific Inc.) in 6-well plates (Sarstedt, EquiMed, Poland) and adhered for 24 hours. At a later stage, the cells were treated with the bilosome suspension. Following 24-hour incubation, the cells were washed twice with phosphate-buffered saline (PBS, BioShop, EPRO, Poland), fixed in 4% paraformaldehyde (Polysciences, Inc., Bergstrasse, Germany) for 10 minutes, and washed again with PBS. The actin cytoskeleton was stained with Alexa Fluor^®^546 Phalloidin following the manufacturer’s protocol (Thermo Fisher Scientific Inc). Fluorshield^TM^ with DAPI (4,6-diamidino-2-phenylindole, fluorescent DNA-binding dye) was applied to visualize the nuclei and mount the cells after excitation at 405 nm. However, the 3D Cell Explorer holotomographic microscope (HTM) was used to analyze cell morphology and data from the microscope were gathered and analyzed using Steve software (Nanolive SA, Sygnis, Poland).

#### Effects on Cell Growth and Survival

In vitro cyto- and photocytotoxicity studies of native RB and co-loaded RB with AST in positively charged bilosomes (in the concentration range for RB from 0.5 µM to 12.5 µM after dilution in the culture medium) were evaluated by the MTT (3-(4,5-dimethylthiazol-2-yl)-2,5-diphenyl tetrazolium bromide) cell proliferation assay (Sigma-Aldrich, Poznan, Poland), as previously reported.^35^ Specifically, the tested cell lines were seeded into 96-well plates (Sarstedt, EquiMed, Wroclaw, Poland) (200 µL each per well – density of almost 2×10^4^ cells). The cytotoxicity tests (without irradiation) were considered after 24 and 72 hours of incubation with analyzed formulations. For the photocytotoxicity studies, the cells were incubated with active compounds or co-loaded bilosomes for 4 hours. After this time, the cells were irradiated for 10 minutes with a total light dose of 10 J/cm^2^ using a HOP 250 lamp (OPTEL Fibre Illuminator, Opole, Poland) equipped with polarized light filtered with barrier filters (λ_max_ 520–560 nm). At the level of the monolayer of cells studied, the energy flux rate was 12 mW/cm^2^. In this case, for the measurements precision, the irradiation dose was verified each time using a light intensity meter for the appropriate wavelength (Optel). The MTT assay used to evaluate the PDT effectiveness was conducted after 24 hours post-irradiation. In each test group, cell viability was expressed as a percentage of cells not exposed to the tested nanocarriers (control group), and absorbance was determined using the GloMax^®^ Discover Microplate Reader (Promega) at 560 nm. The final results rearranged in the graphs were subtracted from the blank and finally normalized to the untreated control. All experiments were performed in triplicate to accurate experiments and measurements.

### Statistical Analysis

The results of the in vitro experiments were presented as mean ± standard deviation (SD) values for a minimum of three replicates, and then compared using a two-way analysis of variance (ANOVA) for multiple comparisons with an alpha level (α) of 0.05. Comparisons of samples exhibiting P-values ≤ 0.05 and ≤ 0.01 were considered statistically significant and highly significant, respectively. All results were analyzed using the commercial software (GraphPad Prism 7.0) and Microsoft Excel.

## Results and Discussion

### Characteristics of the Optimized Bilosomal Formula

Cancer poses a significant public health issue globally and a daily challenge not only for doctors, all medical personnel, or researchers but, above all, the patient and his family. It is a complex disease that develops through a multi-step process, which may include, in particular: (i) resistance to cell death (known as apoptosis), (ii) various changes in cell signaling, (iii) uncontrolled cell growth, as well as (iv) tissue invasion, metastasis to distant sites in the body, and angiogenesis, all of which significantly complicate treatment. According to Global Cancer Incidence, Mortality, and Prevalence (GLOBOCAN), compiled by the International Agency for Research on Cancer (IARC) and distributed as Cancer Today in the Global Cancer Observatory, the number of new cancer cases in the year 2022 exceeded about 19.9 million, with 9.7 million cancer-related deaths. Among them, we distinguish skin cancers, of which melanoma remains the most aggressive and deadly representative, with a steadily increasing incidence (≈332 new cases and ≈ 59 deaths worldwide).^36^,^37^ Therapies targeting melanoma have considerably evolved over the years, and there are now different treatments that are used depending on the stage, grade, or molecular subtype of the tumor. However, the usual clinical options, ie, chemotherapy, immunotherapy, or radiation therapy, are characterized by several drawbacks of anticancer drugs, including limited water solubility, non-specific toxicity, lack of selectivity, or formation of multidrug resistance, which consequently lead to adverse effects on healthy tissues, thereby impairing the human immune system and affecting the patient’s life quality.^38^

Given the above, and considering the risks posed by the scarcity of both alternative and adjuvant treatments targeting melanoma cells, we decided to develop a stimuli-responsive phototherapeutic bilosomal nanoplatform co-encapsulating a second-generation photosensitizer (Rose Bengal, RB) supported by a natural carotenoid (astaxanthin, AST) with antitumor activity (Scheme 1). Several DDS parameters (including size, surface charge, and stability over time), as well as the material composition of the nanocarriers, are among the most essential factors in the design of novel nanostructures for biomedical applications. For this reason, the crucial task was to find the relevant components and optimize the formulation to contain only biocompatible, non-toxic, stable, and spontaneously formed lipid vesicles suitable for subsequent drug-loading studies and their use in photodynamic therapy (PDT) directed against melanoma. In this context, eight types of positively charged structural bilosomes were successfully synthesized and characterized (nanosystems 1–8 included in Supplementary Table 1)^33^. The unique structure of next-generation vesicular systems is composed of (1) the most significant building block of the mammalian cell membrane (phosphatidylcholine, PC), which accounts for as much as 45–55% of all cellular phospholipids;^39^ (2) bile salt-origin biosurfactant (sodium cholate, SC) added to enhance stability, biocompatibility, and penetration properties for possible topical/transdermal delivery of the nanosystem; (3) “stealth” phospholipid polymer (MPEG-2000-DSPE) characterized by biocompatibility, biodegradability, and amphiphilicity, which was also added to the system to increase its stability and improve biodistribution. Targeting cancer cells characterized by a negative charge may be possible through the application of cationic elements, so the non-ionizable cationic lipid (DOTAP), with a well-documented safety profile in numerous clinical trials,^40^ was additionally introduced into the bilosomal structure. Modifying the surface of these nanosystems may also help them interact with the skin, which acts as a negatively charged membrane. This membrane is equipped with a large number of negatively charged lipids in the *stratum corneum* (SC), which will ultimately increase the efficiency of cargo delivery agents when topically administered. As a result, we characterized all the obtained nanostructures by dynamic and electrophoretic light scattering, comparing their average sizes (hydrodynamic diameter, D_H_), polydispersity index (PdI), and surface charge (ζ-potential) depending on their composition. The particle size ranged from 68 to 118 nm. In the case of the studied systems, we noticed a decrease in the size of the carrier after incorporating PEG-lipid into its structure compared to non-PEGylated bilosomes (for details, see Supplementary Table 1).

This phenomenon can be explained by the slightly negative charge of the lipopolymer (DSPE-PEG), which promotes the intensity of lateral repulsion. Repulsive forces contribute to the pushing and curving of the lipid bilayer, which can ultimately diminish the overall diameter of the resulting vesicle. Moreover, embedding PEG derivatives containing lipid molecules in the bilayer of modified liposomes could have led to increased interlayer repulsion, which reduced their lamellar structure.^41–43^ When analyzing the results, it is also possible to notice a reduction in the zeta potential of PEGylated bilosomes compared to formulations not containing the cross-linked DSPE-PEG lipid. It is due to steric shielding, whereby the elastic PEG chains form a hydrated layer on the nanocarrier’s surface, masking its positive charge.^44^,^45^ Finally, for the next stage of our research, we decided to select optimized formulations with the most unimodal size (D_H_ ~ 70 nm) and narrow size distribution (PdI < 0.3), whose physicochemical parameters were unchanged after 30 days of storage (nanosystem 2), which confirmed their high colloidal stability (Supplementary Figure 1).

The optimized formulation was used to co-encapsulate the RB alongside the naturally occurring AST, which ultimately enabled the development of carrier-assisted novel phyto-photodynamic therapy (phyto-PDT) for the treatment of melanoma (Scheme 1). Green light-induced generation of ROS in the presence of RB led to the release of active cargo from bilosomes through membrane permeabilization caused by the possible oxidation of unsaturated lipids and the simultaneous formation of pores in the bilayer of the developed carrier. The production of ROS (specifically singlet oxygen) allows the immune system to be activated, thereby inducing inflammation and an immune response to cancer cells, ultimately leading to the killing of abnormal cells and attacking the tumor’s blood vessels.^46^ Generally, parameters such as size, polydispersity index, and surface charge are the main physicochemical characteristics considered critical quality attributes (CQAs) of the developed nanocarriers.^47^ As shown in Table 1, the submicron D_H_ (about 70 nm), positive ζ-potential around +44 mV, as well as the low PdI (< 0.3), indicating a homogeneous population of RB/AST-loaded bilosomes, allowing them to be considered highly favorable for DDS in further clinical development, especially for topical/transdermal administration.^48^ It stems from the fact that NCs below 100 nm can penetrate SC between corneocytes to a greater extent, thus increasing the uptake of active ingredients encapsulated in NCs in the epidermis and dermis, which is particularly important during skin cancer treatment.^49–51^ It is also worth noting that the zeta potential value of ~ +40 mV is a prerequisite for the high interaction ability of nanostructures with cell membranes.^52^,^53^

**Figure 2:**
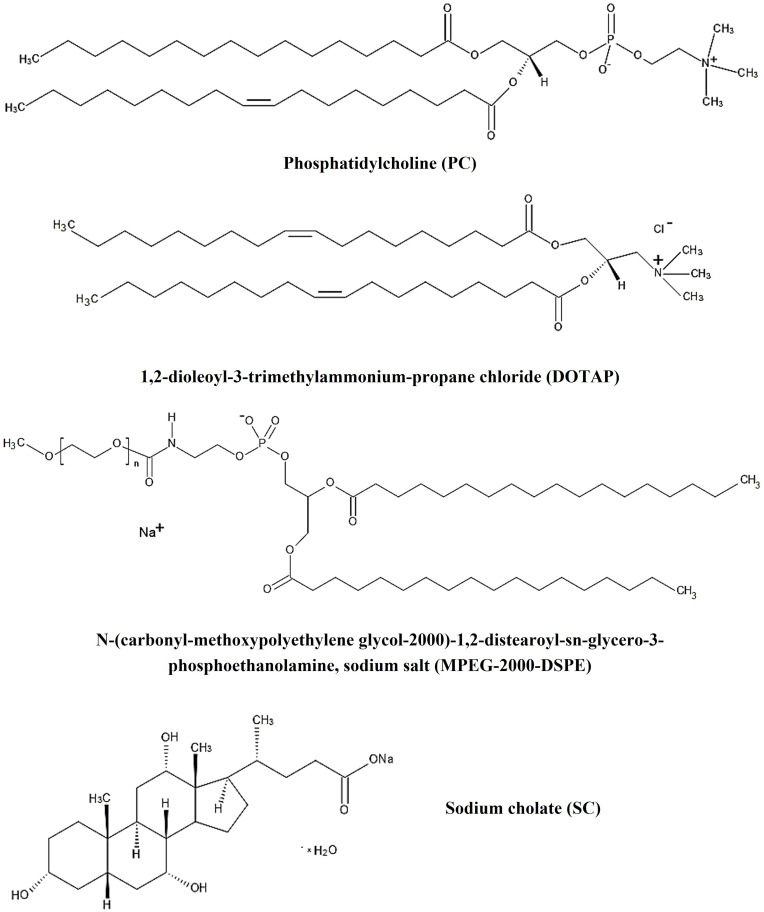
Chemical structure of molecules used to develop positively charged bilosomes.

Transmission electron microscopy (TEM) imaging was performed on empty and co-loaded positively charged bilosomes (Figure 2)

. The images obtained revealed spherical and unilamellar-shaped vesicles of roughly uniform size. In addition, no fusion or increased aggregation of particles was observed in the images found. A crucial achievement in the biomedical application of nanocarriers is the ability to load sufficient amounts of drug needed to achieve therapeutic efficacy. The EE and DL of RB and AST in the positively charged bilosomes were determined via UV-Vis spectroscopy. The spectra of the empty and co-loaded formulations are reported in Supplementary Figure 2. The bilosomal formulation demonstrated high encapsulation efficiency for both active compounds, with entrapment rates above 80% for RB and above 90% for AST (Table 1). The drug loading capacities for these vesicles were 1.1 ± 0.3% and 0.4 ± 0.1%, respectively. These results highlight the efficiency of bilosomes in encapsulating RB and AST, making them a promising candidate for drug delivery applications. The final ratio reflects the maximum loading capacity of the nanoplatform for each drug individually, which was then consistently applied in our subsequent in vitro efficacy studies. In the next step, we performed in vitro release studies of encapsulated active compounds over 72 h using the dialysis method. We evaluated the release rate at various temperatures: 25°C (room temperature), 33°C (skin temperature), and 37°C (body temperature), and under different pH conditions of the release medium: slightly acidic skin pH (5.5) and physiological body pH (7.4). As shown in Supplementary Figure 3, the release of astaxanthin (AST) over 72 hours did not exceed 5%. This low release is likely due to its highly hydrophobic nature, which contributes to a stronger association with the obtained nanocarrier. The release profile of encapsulated Rose Bengal (RB) indicates the nanosystem’s stability over time at physiological pH. However, placing the RB/AST_BIL system in acidic conditions caused the carrier membrane to become permeable to RB, resulting in a higher release rate (approximately 12%) compared to the 4% released at pH 7.4 after 72 hours at 37°C. It is worth noting that the free RB (used as a control) passed rapidly through the dialysis membrane, with approximately 60% of the initial amount found in the release medium within the first few hours. This result highlights the effective role of the positively charged bilosomes in providing a controlled and sustained release, as opposed to the rapid passage of the free drug.

It is important to note that the positive surface charge of the bilosomes did not change significantly after encapsulation of the negatively charged RB, demonstrating the truthful embedding of RB molecules in the aqueous core of the structures. The nanocarriers may change during long-term storage. Therefore, we evaluated the stability of the obtained formulations by monitoring the hydrodynamic diameter (D_H_), polydispersity index (PdI), ζ-potential, and encapsulation efficiency (EE%) for 30 days at 4°C. Table 1

**Table 1:** Physicochemical Properties of Empty (non-loaded) and Double-Loaded Positively Charged Bilosomes

System	t=0 days					t=30 days				
	D_H_^a^ (nm)	PdI^b^	ζ^c^ (mV)	EE^d^_RB_ (%)	EE^d^_AST_ (%)	D_H_^a^ (nm)	PdI^b^	ζ^c^ (mV)	EE^d^_RB_ (%)	EE^d^_AST_ (%)
**Empty Bilosomes**	67 ± 1	0.29 ± 0.01	+45 ± 1	–	–	66 ± 1	0.29 ± 0.00	+45 ± 3	–	–
**RB/AST-loaded Bilosomes**	69 ± 1	0.29 ± 0.01	+44 ± 1	85 ± 3	95 ± 2	71 ± 1	0.29 ± 0.01	+45 ± 1	80 ± 2	91 ± 2

**Abbreviations**: ^a^ D_H_, hydrodynamic diameter; ^b^ PdI, polydispersity index; ^c^ ζ, zeta potential; ^d^ EE, encapsulation efficiency.

shows that we did not observe significant changes in the values of the above parameters throughout the storage period, indicating their high colloidal stability. Considering the rational design of a drug product based on nanocarriers, a crucial element is also the analysis of biological stability, which refers to the integrity of the system under development after it enters the body and, at the same time, the influence of the changing environment on its structure.^54^ To this end, we performed an additional stability assessment, which proved that there were no significant deviations from the behavior of bilosomal vesicles under simulated environmental conditions (incubation of the formulation in PBS at physiological pH 7.4, tumor pH 6.5, skin pH 5.5, and DMEM without or with 10% FBS) (for details, see Supplementary Figure 4).

### Singlet Oxygen Photogeneration

**Figure 3:**
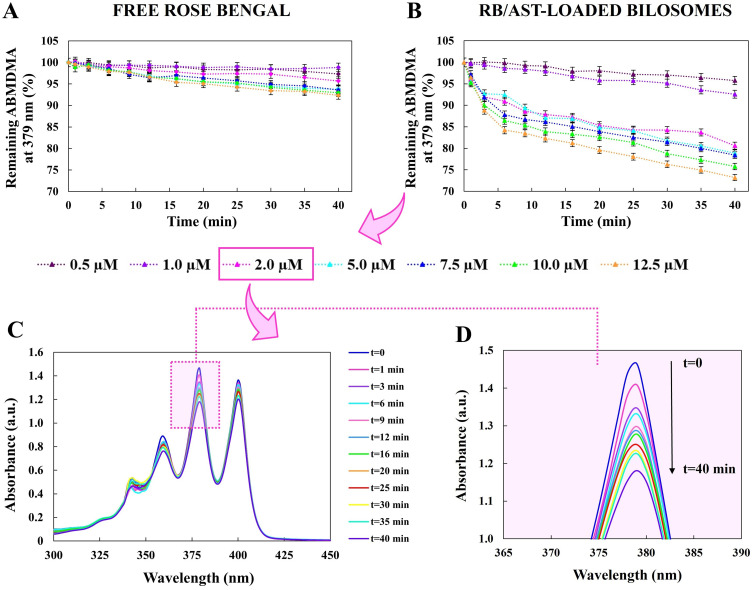
Photooxidation of 0.15 mM ABMDMA by ^1^O_2_ generated by Rose Bengal water solution (**A**) and Rose Bengal supported by astaxanthin co-encapsulated in the positively charged bilosomes (**B**) in the concentration range for photosensitizer from 0.5 µM to 12.5 µM. The absorption spectrum of ABMDMA solution containing double-loaded bilosomes (at a final RB concentration of 2 µM) as a function of green light irradiation times (0 to 40 minutes) (**C**) along with the enlarged area of crucial spectral changes at 379 nm (λ_max_ of ABMDMA) (**D**).

Molecular oxygen in the excited singlet state (^1^O_2_) is one of the first molecules formed under aerobic conditions instantly after exposure to light of a specific wavelength during photodynamic therapy (PDT), leading to direct oxidation of surrounding bio-molecules, shutting down the circulatory system, and ultimately an inflammatory or immune response to the tumor tissue.^55^ Accordingly, exploring the dynamics of the ^1^O_2_ generation of novel nanophotosensitizers is especially relevant for their subsequent application in biological and medical research. During our study, we used 9,10-anthracenediyl-bis(methylene)dimalonic acid (ABMDMA) as a spectrophotometric probe to detect the ^1^O_2_ generation by the hydrophilic photosensitizer (Rose Bengal, RB) and RB supported by a hydrophobic natural anticarcinogenic ingredient (astaxanthin, AST) co-encapsulated in positively charged bilosomes. The ABMDMA compound shows an ordered absorption spectrum, with maxima at around 380 nm, and its characteristic absorption undergoes photobleaching due to the interaction of ^1^O_2_ and the endoperoxide adduct’s formation. The time-dependent decrease in the detection agent absorption at 379 nm (λ_max_ of ABMDMA) was monitored spectrophotometrically in the presence of individual samples over a wide range of photosensitizer concentrations (0.5–12.5 µM), as shown in Figure 3.

According to the data shown in Figure 3, the activity of the ^1^O_2_ scavenger molecule gradually decreased after irradiating the samples with green light in the 520–560 nm range. Significantly, non-encapsulated RB showed a lower capacity to generate singlet oxygen (Figure 3A) than the photosensitizer co-loaded with AST (Figure 3B). Considerable effect of the RB/AST-loaded bilosomes was evident already at a concentration of 2 µM (Figure 3C and D). At the same time, the activity of free RB was negligible, indicating that the encapsulated second-generation PS can be applied potentially at lower doses necessary for effective PDT. We emphasize that ABMDMA photobleaching is approximately 4-fold greater for RB/AST co-loaded bilosomes compared to free RB for a concentration of 2 µM (Figure 3D), which is a functional proof of the gain of the carrier-mediated in the singlet oxygen (ROS) generation. In this case, light irradiation reduced the absorbance of ABMDMA by approximately 5 and 20% of its original value after 40 minutes for RB aqueous solution and RB/AST-loaded bilosomes, respectively. Such differences may be due to the tendency of organic dyes (including just RB) to form aggregations into dimers or higher multimers in aqueous solution, an unfavorable phenomenon that limits their widespread use in PDT due to their impaired photochemical response. Photoinduced degradation of dyes can reduce the ability to generate ROS and consequently their lower efficacy against target cells (ie, cancer cells).^56^,^57^ Therefore, for this purpose, we simultaneously carried out additional experiments for evaluating the photobleaching of Rose Bengal, which showed that the encapsulated dye exhibited better photostability during exposure to green light than its non-encapsulated molecules (for details, see Supplementary Figure 5). It is also worth noting that the empty positively charged bilosomes did not produce ^1^O_2_ - only a reduction in ABMDMA content of 0.5% to 4% of the initial value after 40 minutes of exposure to green light was observed, depending on the sample concentration used (Supplementary Figure 6). From the presented charts, it can be concluded that encapsulation of photosensitizer in bilosomal nanosystem led to an increase in the ^1^O_2_ generating capacity and thus PDT enhancement compared to the same photosensitizer in an aqueous medium without nanocarriers under control conditions.

### Quantification and Bioimaging of Cellular Uptake

**Figure 4:**
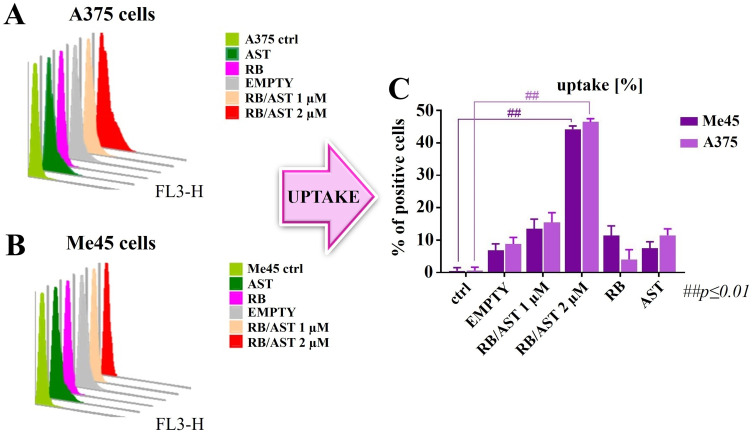
Flow cytometry histograms for the uptake of studied nanosystems by human melanoma A375 (**A**) and Me45 (**B**) cell lines with the FACS uptake comparison for both cell lines (**C**) after 24 hours incubation at 37°C, ## statistical analysis: p ≤ 0.01.

Apart from generating singlet oxygen, cellular uptake of active compounds is an additional factor in successful PDT, which requires precise therapeutic interventions. Taking advantage of the specific morphological properties of cancer cells (including the significantly higher negative surface charge compared to normal cells), it is possible to develop individual carrier systems that target cells. Due to an imbalance in enzyme activity (ie, flippase, floppase, and scramblase) induced by oxidative stress, cancer cells overexpress negatively charged phospholipids - phosphatidylserine (PtdSer) and phosphatidylethanolamine (PE). Moreover, rapid cell division contributes to a very high metabolic rate in these cells, leading to the Warburg effect (ie, excessive production of lactic acid, transported from cancer cells in the form of lactate anions), which also affects their negative surface charge on the outer membrane. Thus, introducing positive charge-driven components into the carrier structure can be an effective strategy for significantly enhancing the uptake and accumulation of particular therapeutic agents against cancer cells.^58–60^ Figure 4 shows the percentage of cells positive for uptake of free and encapsulated RB and AST in positively charged bilosomes against two melanoma cancer cell lines: A375 (light purple) and Me45 (dark purple), with the corresponding histograms. Empty nanocarriers were used as a negative control and did not cause significant cellular uptake, and control groups (ctrl) were present for each of the cell lines tested. Multifunctional bilosomes were analyzed at two concentrations, concerning the final RB concentration (1 µM and 2 µM). Our study showed a higher percentage of positive cells for RB/AST-loaded bilosomes (for both concentrations tested) compared to non-encapsulated compounds active against Me45 cells. This effect is highly dose-dependent. There is an almost 3-fold increase in uptake for concentrations of 2 µM compared to 1 µM. We observed the same pattern for the A375 cell line - in this case, the percentage of positive cells is slightly higher compared to Me45 for both concentrations of multifunctional bilosomes. Both free (non-loaded) AST and RB molecules showed minimal uptake in the two cell lines. Statistical analysis verified that the differences in cellular uptake were significant (p≤ 0.01) compared to controls in the Me45 and A375 cell lines. The lipophilic nature of PS is crucial to its biodistribution and cellular localization, determining its therapeutic efficacy. The poor lipophilicity and anionic nature of non-encapsulated RB lead to poor cellular uptake and reduced permeation through the cell membrane, resulting in insufficient cellular accumulation at low concentrations of this photosensitizer in the absence of special nanocarriers.^61^,^62^ So, the increased cellular uptake for the RB/AST-loaded bilosomes is arguably due to the shielding of the negatively charged groups present in the RB structure by the positive surface charge of the formulated bilosomes. Therefore, by creating a favorable internal environment, positively charged nanocarriers help overcome both the poor solubility of the dye in lipids and also lead to much more efficient intracellular transport, which is fundamental to downstream mechanisms of programmed cell death.^63^

**Figure 5:**
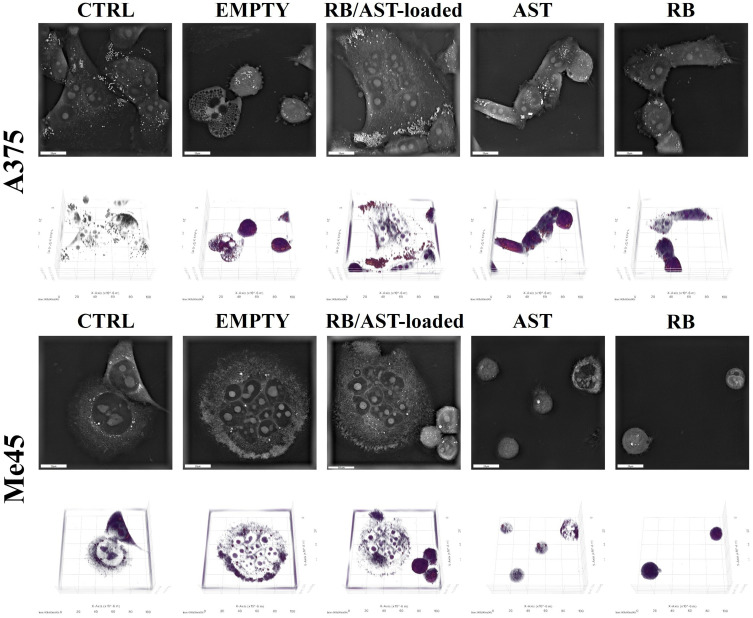
Bioimaging study in A375 (upper panel) and Me45 (lower panel) cells after treatment with empty and RB/AST-co-loaded bilosomes, as well as free active compounds (AST and RB), with the final photosensitizer (PS) concentration set to 2 µM. For each condition, corresponding 2D X-Y slice images (up) and 3D reconstructions (down) provide a comprehensive view of cellular responses to the treatments. The scale bar represents 20 µm.

**Figure 6:**
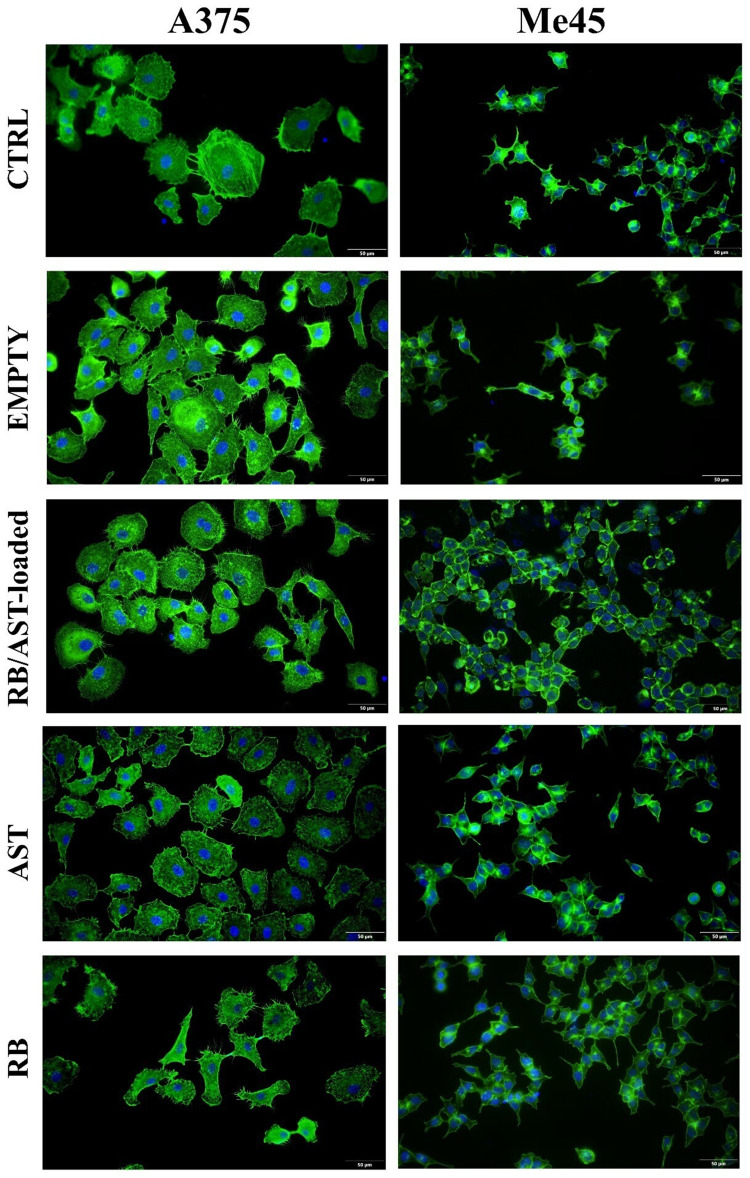
Immunofluorescence F-actin staining performance on human melanoma A375 and Me45 cells after treatment with empty and co-loaded bilosomes, as well as free active compounds. The final photosensitizer (Rose Bengal, RB) concentration was 2 µM. The scale bar represents 50 µm.

In the next step, we evaluated the effects of empty and loaded positively charged bilosomes on the morphology of human melanoma skin cancer cells (A375 and Me45). The in vitro bioimaging results are shown in Figures 5 and 6. We first presented 3D cell bioimaging performed using holotomographic microscopy (HTM), showing cell morphology after exposure to the bilosomes and the free active cargo (Figure 5). The primary advantage of holotomographic imaging is the three-dimensional illustration and the ability to determine whether nanocarriers are accumulating in the cell.^64^ In A375 cells, distinct morphological changes are evident. The control (CTRL) and empty bilosome (EMPTY) groups show typical cellular morphology without significant alterations. In the case of cells treated with RB/AST-loaded bilosomes, there is an observable slight increase in cell size, along with the accumulation of lysosomes and lipid droplets, particularly in the peripheral regions of the cells (a little larger than in the case of control cells). This peripheral accumulation may indicate a response to the loaded bilosome treatment, potentially related to increased cellular stress or altered metabolic processes.

On the other hand, the free AST and RB compounds provoked evident cell shrinkage, suggesting these active compounds can influence cell morphology. In Me45 melanoma cells, HTM imaging revealed similar morphological changes across treatments compared to A375 cells. Control cells maintain regular morphology. The exposure to empty or loaded bilosomes stimulated cells’ aggregation and a visible increase in organelle density near the peripheral region, with noticeable clustering of lysosomes and lipid droplets. This aggregation can be explained as a protective mechanism, where cells cluster together to minimize individual exposure and potentially enhance cell-cell signaling. This aggregation could also help to concentrate defensive responses, such as lysosomal activity. The exposure to AST or RB caused significant cell shrinkage, reduced size, and altered morphology, indicating that the native (non-loaded) hybrid cargo can be toxic and causes morphological disorders to a much greater extent than its encapsulated forms. Interestingly, in both cell lines, similar effects of the native cargo were observed. The more significant cell aggregation observed after the Me45 cells incubated with the studied nanocarriers can be explained by several potential mechanisms, eg, cellular stress response, membrane interaction with bilosomes, and endocytosis or lipid metabolism activation.^65^ The intensity of such stress determines whether the cell will enter the apoptosis pathway or be restored to homeostasis.^66^ Additionally, the presence of bilosomes might also disrupt normal intracellular trafficking, leading to organelle accumulation in specific regions, such as the cell periphery. This accumulation of organelles, including lysosomes and lipid droplets, could be the effect of bilosomes’ interference with the cytoskeletal or motor proteins, which are responsible for organelle movement.

We then carried out F-actin staining to further analyze the effects of the engineered nanocarriers on cellular structures (ie, the cytoskeleton). As shown in Figure 6, the obtained bilosomes did not affect the dynamics of the cytoskeleton, as we did not observe any significant reorganization of the active fibers after exposure to the engineered nanostructures, either empty or charge-loaded with positively charged bilosomes. In the case of free RB molecules in the A375 line, we observe some decrease in the number of cells, as well as cell elongation and deformation, indicating the toxic effect of the unloaded photosensitizer. Overall, the above results showed that the novel positively charged formulations did not directly affect cell morphology or the dynamics of the activated cytoskeleton, confirming their high biocompatibility - one of the most crucial features of the newly developed biomaterials for their further potential biomedical applications. Moreover, it is known that other cationic colloidal nanocarriers smaller than approximately 100 nm (such as our 70 nm RB/AST bilosomes) can effectively penetrate tumour spheroids and other dense extracellular matrices.^67^ The addition of a PEG lipid (MPEG-DSPE2000) further reduces aggregation and improves movement through interstitial spaces, reinforcing the platform’s suitability for three-dimensional tissue.^68^,^69^

### In Vitro Dark Cytotoxicity and Photodynamic Action

The final stage of our research was based on the analysis of the cytotoxicity and phyto-photodynamic activity of the obtained positively charged bilosomes against human melanoma cells derived from an expansion culture of a solid tumor of a 54-year-old woman (A375) and human malignant melanoma cells derived from cutaneous melanoma metastasis to lymph nodes (Me45). It is worth noting that cytocompatibility was also evaluated on primary human cutaneous keratinocytes (HaCaT), used as a control line, due to the possibility of using the nanocarriers in topical/transdermal delivery of active ingredients. Firstly, the cytotoxicity of empty and RB/AST-loaded bilosomes was analyzed over a wide range of RB photosensitizer concentrations (from 0.5 µM to 12.5 µM) and expressed as mitochondrial dehydrogenase activity (MTT cell proliferation assay). Cell viability studies, estimated after a 24-hour incubation in the dark of the bilosomal formulation (Supplementary Figure 7), demonstrated the safety of the tested nanocarriers over the entire concentration range for the A375 and Me45 cell lines. Moreover, for extended incubation times of up to 72 hours, we constantly observed a high degree of the nanostructures safety against A375 cells in the concentration range tested, even for the highest 12.5 µM. For the Me45 cell line, in contrast, we observed the opposite effect after a 72-hour incubation. This phenomenon may have been caused by a reduction in mitochondrial dehydrogenase activity, resulting in a significant decrease in cell viability, probably due to induced accumulation of lysosomes (ie, lysosomal stress response, LSR), considered an early marker of the autophagy process. The level of intensity of the LSR, can affect the behavior of a cell - it can either lead to cell death or restore homeostasis. The stress levels during the first 72 hours may have reached a critical threshold for Me45 cells, causing activation of programmed cell death pathways. A375 cells, on the other hand, may have exhibited a multifaceted response to the onset of lysosomal stress, including, in particular, (i) the initiation of a set of defense mechanisms aimed at alleviating the effects of the occurring stress (ie, regulation of lysosomal biogenesis, engagement of antioxidant defenses to counter stress-induced ROS, and activation of autophagy pathways to remove damaged organelles) and (ii) gradual alleviation of the initial lysosomal stress and transition to a state of cellular homeostasis.^66^ The obtained results are largely compatible with the most evident morphological changes, as cell aggregation or lysosome accumulation, observed by HTM bioimaging of Me45 cells in comparison to the A375 cell line. Nevertheless, before targeting photodynamic activity, we also checked the cytocompatibility of the nanoplatform against the HaCaT cell line. The incubation time-dependent cytotoxicity for a wide range of concentrations (0.5 µM to 12.5 µM) is shown in Supplementary Figure 8. Complementary experiments showed the safety of the formulations used for human skin cells up to a concentration of 2 µM (inclusive), reaching more than 70% of control cells even after extended incubation times of up to 72 hours. These studies proved that the formulations did not change the metabolic conditions of the HaCaT culture and, as a result, allowed us to choose the highest and, at the same time, the most beneficial and safe for normal cell dilution of the bilosomal sample (ultimately equal to the final concentration of 2 µM of loaded RB) to examine the photodynamic activity.

**Figure 7:**
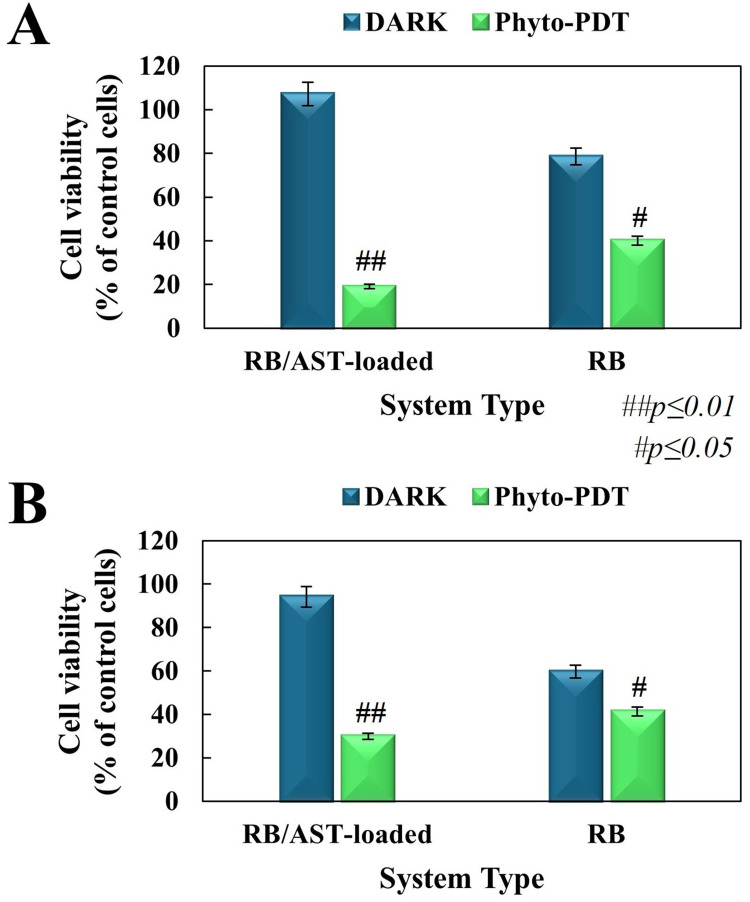
The dark cytotoxicity (without irradiation) and photocytotoxicity (phyto-PDT action) post-green light irradiation of double-loaded bilosomes compared to free RB molecules (the final PS concentration was 2 µM) upon human melanoma A375 (**A**) and Me45 (**B**) cell lines. The cell viability in each group was expressed as a percentage of the control (untreated with the studied nanocarriers) cancer cells. The results are represented as mean values ± SD for minimum n = 3, ## p ≤ 0.01, # p ≤ 0.05.

The PDT activity of the co-loaded hybrid cargo was evaluated on both melanoma cancer cells exposed to green light (10 min with a total light dose of 10 J/cm^2^, λ_max_ 520–560 nm), followed by a 24-hour incubation. The results in Figure 7 show a significant decrease after irradiation of the remaining viable cells for both cultures tested compared to untreated cancer cells and free RB molecules. Reducing cellular mitochondrial activity proved more effective in the A375 cell line, causing a decrease in cell viability to less than 20% of the control. The observed phototoxic effect was almost twice as strong as compared to free (non-loaded) RB molecules. The photodynamic action in the Me45 cell line was slightly less significant (where the decrease in cell viability remained below 30% of the control), but still better than in the case of the free photosensitizer. Such trends are in good correlation with the ROS generation studies presented above (see *Singlet Oxygen Photogeneration*). The results indicated the higher efficiency in ROS production for the co-loaded photosensitizer, proving the advantage of its encapsulation in the obtained nanoplatform. Interestingly, the photocytotoxicity effect was not observed with the HaCaT cell line, even when a longer (up to 72 hours) incubation time was used (Supplementary Figure 9). Under identical illumination, the RB/AST‑bilosomes reduced melanoma viability to <30% while ≥80% of HaCaT cells (Figure 7 and Supplementary Figure 9, respectively). Together with the higher uptake observed in melanoma (Figure 4), these data strongly suggest a preferential action on cancer cells within a mixed population. Mechanistically, the positive ζ‑potential (+44 mV) of the bilosomes favours electrostatic interaction with the more negatively charged melanoma membrane, whereas normal keratinocytes internalize the carrier less efficiently. Consequently, the performed studies showed the enhanced PDT activity of the co-loaded hybrid cargo, which goes hand in hand with its photocytotoxicity upon human melanoma cells as well as the nanoplatform protective effect upon normal skin cells even after the irradiation process.

## Conclusion

In the following paper, we rationally constructed a green light-triggered multifunctional nanoplatform, which consisted of DOTAP-modified bilosomes that co-loaded second-generation photosensitizer (Rose Bengal, RB) in the aqueous core and naturally anticancer compounds (astaxanthin, AST) in the bilayer membrane. Physicochemical characteristics of the double-loaded bilosomes using DLS, ELS, and TEM showed that they have a nanometric size (D_H_ ~ 70 nm), positive surface charge (+44 mV), spherical morphology, and high colloidal stability. The results showed that it is possible to enhance the ability to generate reactive oxygen species by encapsulating the photosensitizer in the carrier structure, which prevents its rapid photodegradation. The internalization properties were evaluated against human skin epithelial (A375) and malignant (Me45) melanoma cell lines. Our results evidenced an increased uptake of multifunctional bilosomes compared to non-encapsulated biologically active molecules. On the other hand, holotomography and fluorescence results showed no significant effect on the non-treated cells, unlike the free RB or AST forms, where we could see other principal morphological changes and a reduction in cell number. Nanocarriers containing RB and simultaneously supported with AST exhibited high phyto-photodynamic activity induced by green light (primarily in the A375 cell line), confirmed by a decrease in the cell mitochondrial activity. Considering the presented findings, the developed next-generation bilosomal positively charged formulation represents a promising approach to combating melanoma skin cancer.

Our current work offers preliminary physicochemical and photodynamic validation of the nanoplatform for transdermal drug delivery. While the use of conventional 2D cell cultures is a known limitation that does not fully replicate the complexity of the tumor microenvironment, these models constitute an essential first stage in providing valuable insights into the efficacy and potential toxicity of therapeutic agents. To fully realize its potential, future research should focus on investigating mechanistic endpoints using advanced models such as in vitro three-dimensional (3D) organotypic melanoma spheroids and in vivo models. Moreover, to fully assess the nanoplatform’s potential for transdermal application, permeability assays in vertical Franz diffusion cells with specialized skin-simulating membranes are essential for a comprehensive understanding of the nanoplatform’s transdermal delivery.

## Abbreviations

^1^O_2_, singlet oxygen; A375, human epithelial skin melanoma cell line; ABMDMA, 9,10-anthracenediyl-bis(methylene)dimalonic acid; APIs, active pharmaceutical ingredients; AST, astaxanthin; BS, bile salts; CHCl_3_, chloroform; Chol, cholesterol; CQAs, critical quality attributes; DDSs, drug delivery systems; D_H_, hydrodynamic diameter; DLS, dynamic light scattering; DMEM, Dulbecco’s Modified Eagle Medium; DMSO, dimethyl sulfoxide; DOTAP, 1,2-dioleoyl-3-trimethylammonium-propane, chloride; EE, encapsulation efficiency; ELS, electrophoretic light scattering; FBS, fetal bovine serum; FC, flow cytometry; FM, fluorescence microscope; HaCaT, human keratinocyte cell line; HTM, holotomographic microscope; LSR, lysosomal stress response; Me45, human malignant melanoma cell line; MMP, matrix metalloproteinase; MPEG-2000-DSPE, N-(carbonyl-methoxypolyethylene glycol-2000)-1,2-distearoyl-sn-glycero-3-phosphoethanolamine, sodium salt; MTT, 3-(4,5-dimethylthiazol-2-yl)-2,5-diphenyl tetrazolium bromide; NCs, nanocarriers; PBS, phosphate-buffered saline; PC, phosphatidylcholine; PdI, polydispersity index; PDT, photodynamic therapy; PE, phosphatidylethanolamine; phyto-PDT, phyto-photodynamic therapy; PLs, phospholipids; PS, photosensitizer; PtdSer, phosphatidylserine; RB, Rose Bengal; ROS, reactive oxygen species; SC, sodium cholate; SC, *stratum corneum*; TEM, transmission electron microscopy; HF, tetrahydrofuran; UV-Vis, ultraviolet-visible spectroscopy.
